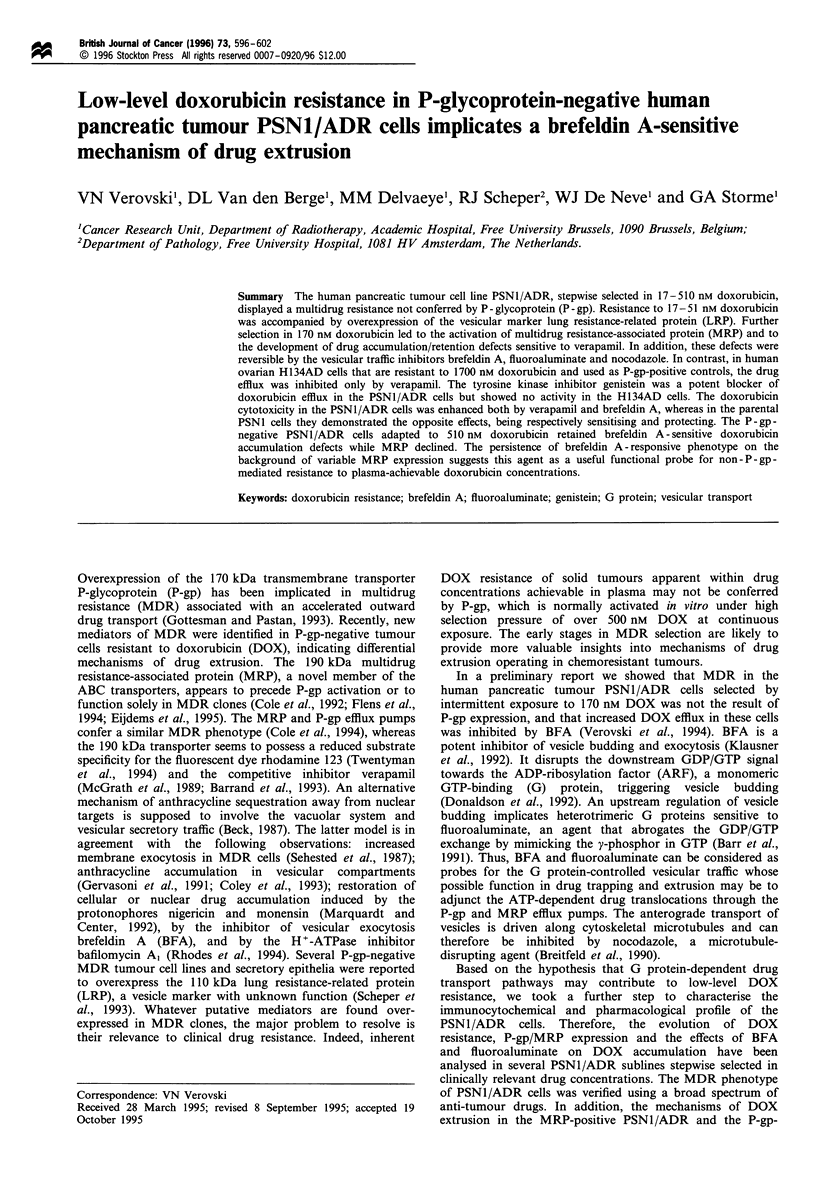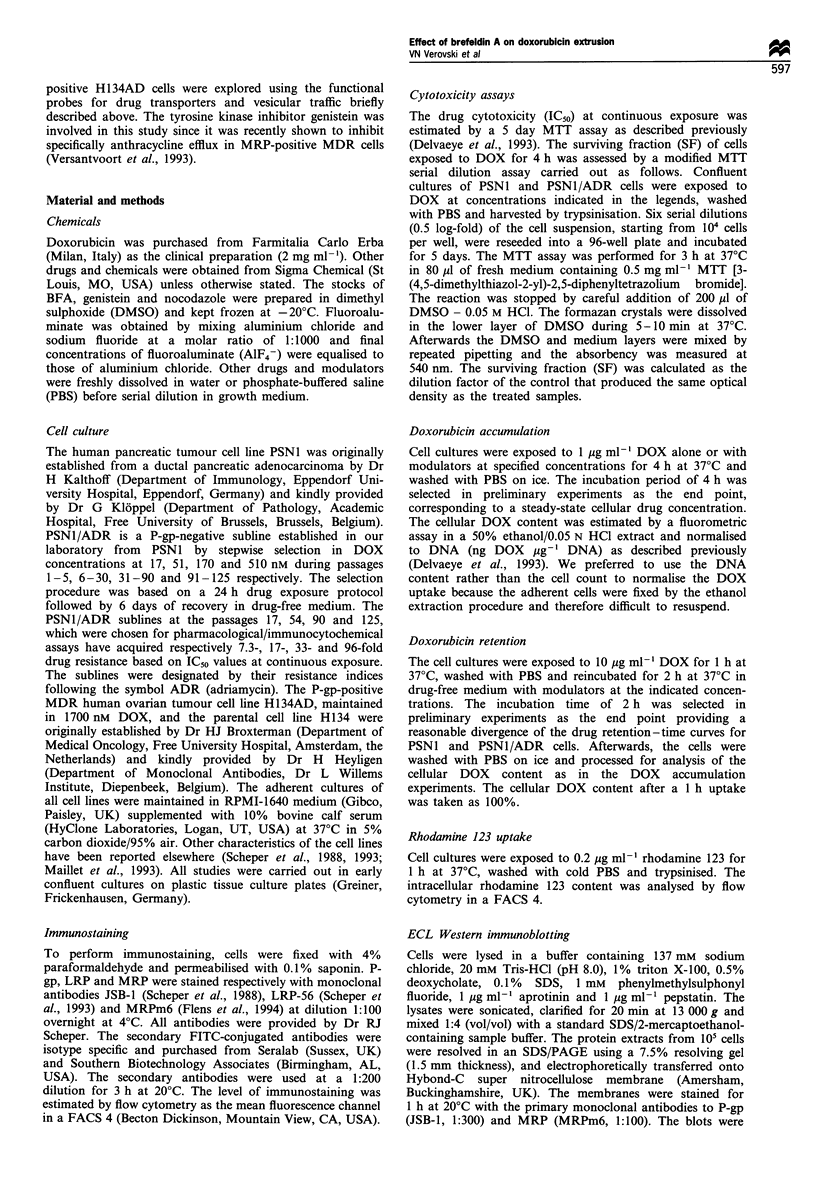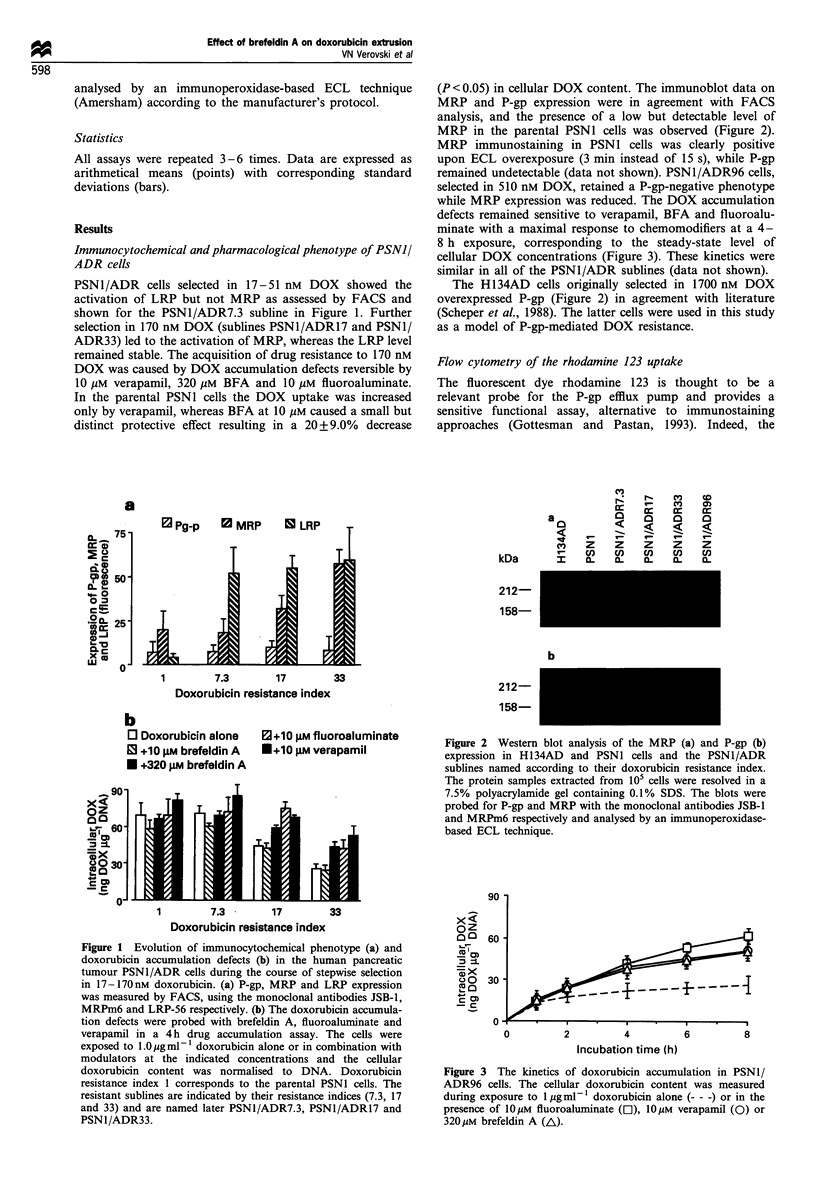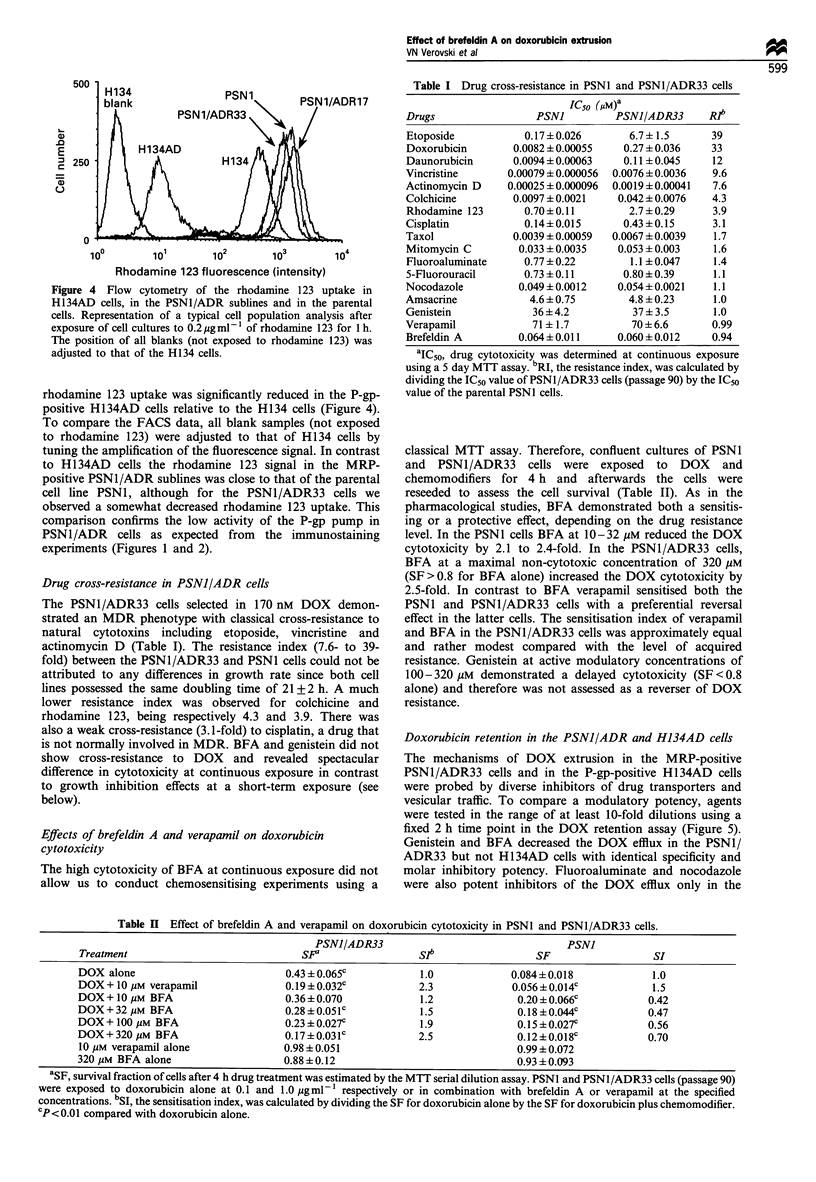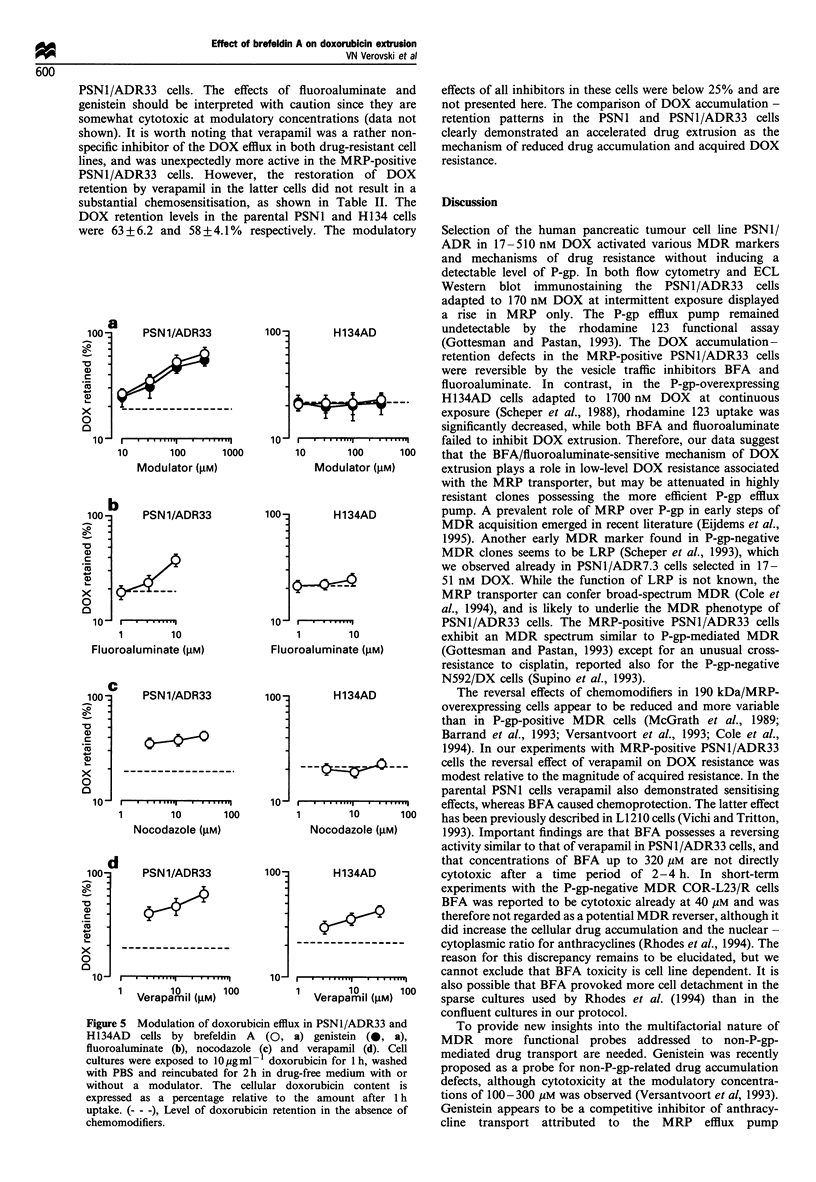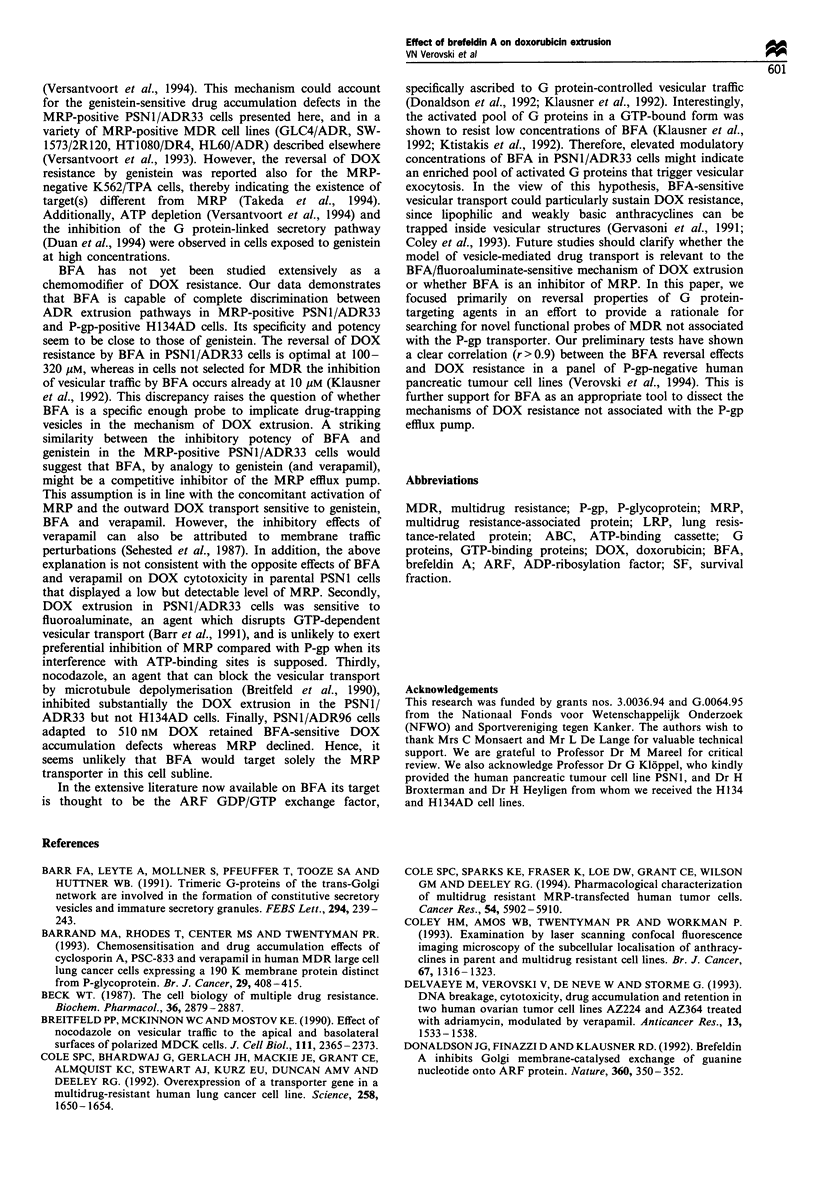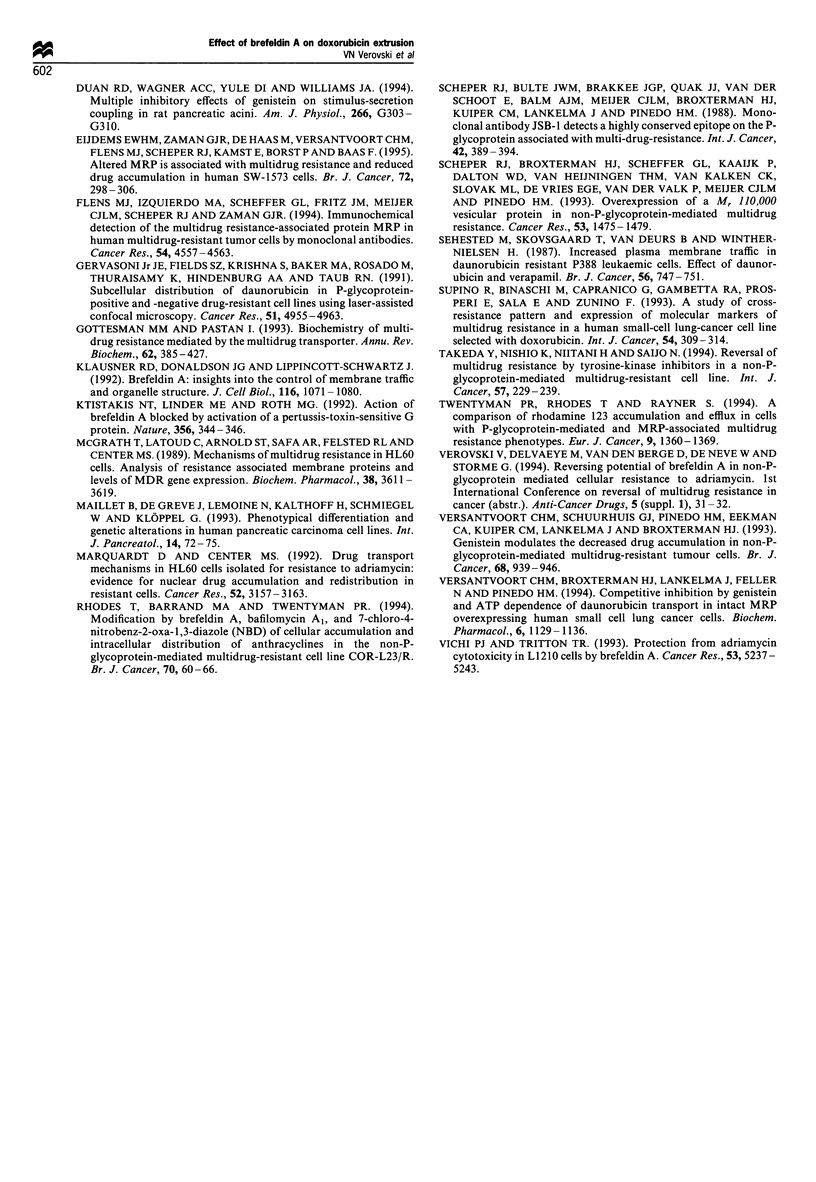# Low-level doxorubicin resistance in P-glycoprotein-negative human pancreatic tumour PSN1/ADR cells implicates a brefeldin A-sensitive mechanism of drug extrusion.

**DOI:** 10.1038/bjc.1996.103

**Published:** 1996-03

**Authors:** V. N. Verovski, D. L. Van den Berge, M. M. Delvaeye, R. J. Scheper, W. J. De Neve, G. A. Storme

**Affiliations:** Cancer Research Unit, Department of Radiotherapy, Academic Hospital, Free University Brussels, Belgium.

## Abstract

**Images:**


British Journal of Cancer (1996) 73, 596-602
© 1996 Stockton Press All rights reserved 0007-0920/96 $12.00

### Low-level doxorubicin resistance in P-glycoprotein-negative human pancreatic tumour PSN1/ADR cells implicates a brefeldin A-sensitive mechanism of drug extrusion

VN Verovski[1], DL Van den Berge[1], MM Delvaeye[1], RJ Scheper[2], WJ De Neve[1] and GA Storme[1]

[1]*Cancer Research Unit, Department of Radiotherapy, Academic Hospital, Free University Brussels, 1090 Brussels, Belgium;* [2]*Department of Pathology, Free University Hospital, 1081 HV Amsterdam, The Netherlands.*

**Summary** The human pancreatic tumour cell line PSN1/ADR, stepwise selected in 17-510 nM doxorubicin, displayed a multidrug resistance not conferred by P-glycoprotein (P-gp). Resistance to 17-51 nM doxorubicin was accompanied by overexpression of the vesicular marker lung resistance-related protein (LRP). Further selection in 170 nM doxorubicin led to the activation of multidrug resistance-associated protein (MRP) and to the development of drug accumulation/retention defects sensitive to verapamil. In addition, these defects were reversible by the vesicular traffic inhibitors brefeldin A, fluoroaluminate and nocodazole. In contrast, in human ovarian H134AD cells that are resistant to 1700 nM doxorubicin and used as P-gp-positive controls, the drug efflux was inhibited only by verapamil. The tyrosine kinase inhibitor genistein was a potent blocker of doxorubicin efflux in the PSN1/ADR cells but showed no activity in the H134AD cells. The doxorubicin cytotoxicity in the PSN1/ADR cells was enhanced both by verapamil and brefeldin A, whereas in the parental PSN1 cells they demonstrated the opposite effects, being respectively sensitising and protecting. The P-gp-negative PSN1/ADR cells adapted to 510 nM doxorubicin retained brefeldin A-sensitive doxorubicin accumulation defects while MRP declined. The persistence of brefeldin A-responsive phenotype on the background of variable MRP expression suggests this agent as a useful functional probe for non-P-gp-mediated resistance to plasma-achievable doxorubicin concentrations.

**Keywords:** doxorubicin resistance; brefeldin A; fluoroaluminate; genistein; G protein; vesicular transport

Overexpression of the 170 kDa transmembrane transporter P-glycoprotein (P-gp) has been implicated in multidrug resistance (MDR) associated with an accelerated outward drug transport (Gottesman and Pastan, 1993). Recently, new mediators of MDR were identified in P-gp-negative tumour cells resistant to doxorubicin (DOX), indicating differential mechanisms of drug extrusion. The 190 kDa multidrug resistance-associated protein (MRP), a novel member of the ABC transporters, appears to precede P-gp activation or to function solely in MDR clones (Cole *et al.*, 1992; Flens *et al.*, 1994; Eijdems *et al.*, 1995). The MRP and P-gp efflux pumps confer a similar MDR phenotype (Cole *et al.*, 1994), whereas the 190 kDa transporter seems to possess a reduced substrate specificity for the fluorescent dye rhodamine 123 (Twentyman *et al.*, 1994) and the competitive inhibitor verapamil (McGrath *et al.*, 1989; Barrand *et al.*, 1993). An alternative mechanism of anthracycline sequestration away from nuclear targets is supposed to involve the vacuolar system and vesicular secretory traffic (Beck, 1987). The latter model is in agreement with the following observations: increased membrane exocytosis in MDR cells (Sehested *et al.*, 1987); anthracycline accumulation in vesicular compartments (Gervasoni *et al.*, 1991; Coley *et al.*, 1993); restoration of cellular or nuclear drug accumulation induced by the protonophores nigericin and monensin (Marquardt and Center, 1992), by the inhibitor of vesicular exocytosis brefeldin A (BFA), and by the $H^+$-ATPase inhibitor bafilomycin $A_1$ (Rhodes *et al.*, 1994). Several P-gp-negative MDR tumour cell lines and secretory epithelia were reported to overexpress the 110 kDa lung resistance-related protein (LRP), a vesicle marker with unknown function (Scheper *et al.*, 1993). Whatever putative mediators are found over-expressed in MDR clones, the major problem to resolve is their relevance to clinical drug resistance. Indeed, inherent DOX resistance of solid tumours apparent within drug concentrations achievable in plasma may not be conferred by P-gp, which is normally activated *in vitro* under high selection pressure of over 500 nM DOX at continuous exposure. The early stages in MDR selection are likely to provide more valuable insights into mechanisms of drug extrusion operating in chemoresistant tumours.

In a preliminary report we showed that MDR in the human pancreatic tumour PSN1/ADR cells selected by intermittent exposure to 170 nM DOX was not the result of P-gp expression, and that increased DOX efflux in these cells was inhibited by BFA (Verovski *et al.*, 1994). BFA is a potent inhibitor of vesicle budding and exocytosis (Klausner *et al.*, 1992). It disrupts the downstream GDP/GTP signal towards the ADP-ribosylation factor (ARF), a monomeric GTP-binding (G) protein, triggering vesicle budding (Donaldson *et al.*, 1992). An upstream regulation of vesicle budding implicates heterotrimeric G proteins sensitive to fluoroaluminate, an agent that abrogates the GDP/GTP exchange by mimicking the γ-phosphor in GTP (Barr *et al.*, 1991). Thus, BFA and fluoroaluminate can be considered as probes for the G protein-controlled vesicular traffic whose possible function in drug trapping and extrusion may be to adjunct the ATP-dependent drug translocations through the P-gp and MRP efflux pumps. The anterograde transport of vesicles is driven along cytoskeletal microtubules and can therefore be inhibited by nocodazole, a microtubule-disrupting agent (Breitfeld *et al.*, 1990).

Based on the hypothesis that G protein-dependent drug transport pathways may contribute to low-level DOX resistance, we took a further step to characterise the immunocytochemical and pharmacological profile of the PSN1/ADR cells. Therefore, the evolution of DOX resistance, P-gp/MRP expression and the effects of BFA and fluoroaluminate on DOX accumulation have been analysed in several PSN1/ADR sublines stepwise selected in clinically relevant drug concentrations. The MDR phenotype of PSN1/ADR cells was verified using a broad spectrum of anti-tumour drugs. In addition, the mechanisms of DOX extrusion in the MRP-positive PSN1/ADR and the P-gp-

Correspondence: VN Verovski
Received 28 March 1995; revised 8 September 1995; accepted 19 October 1995

positive H134AD cells were explored using the functional probes for drug transporters and vesicular traffic briefly described above. The tyrosine kinase inhibitor genistein was involved in this study since it was recently shown to inhibit specifically anthracycline efflux in MRP-positive MDR cells (Versantvoort *et al*., 1993).

### Material and methods

*Chemicals*

Doxorubicin was purchased from Farmitalia Carlo Erba (Milan, Italy) as the clinical preparation (2 mg $ml^{-1}$). Other drugs and chemicals were obtained from Sigma Chemical (St Louis, MO, USA) unless otherwise stated. The stocks of BFA, genistein and nocodazole were prepared in dimethyl sulphoxide (DMSO) and kept frozen at −20°C. Fluoroaluminate was obtained by mixing aluminium chloride and sodium fluoride at a molar ratio of 1:1000 and final concentrations of fluoroaluminate ($AlF_4^-$) were equalised to those of aluminium chloride. Other drugs and modulators were freshly dissolved in water or phosphate-buffered saline (PBS) before serial dilution in growth medium.

*Cell culture*

The human pancreatic tumour cell line PSN1 was originally established from a ductal pancreatic adenocarcinoma by Dr H Kalthoff (Department of Immunology, Eppendorf University Hospital, Eppendorf, Germany) and kindly provided by Dr G Klöppel (Department of Pathology, Academic Hospital, Free University of Brussels, Brussels, Belgium). PSN1/ADR is a P-gp-negative subline established in our laboratory from PSN1 by stepwise selection in DOX concentrations at 17, 51, 170 and 510 nM during passages 1−5, 6−30, 31−90 and 91−125 respectively. The selection procedure was based on a 24 h drug exposure protocol followed by 6 days of recovery in drug-free medium. The PSN1/ADR sublines at the passages 17, 54, 90 and 125, which were chosen for pharmacological/immunocytochemical assays have acquired respectively 7.3-, 17-, 33- and 96-fold drug resistance based on $IC_{50}$ values at continuous exposure. The sublines were designated by their resistance indices following the symbol ADR (adriamycin). The P-gp-positive MDR human ovarian tumour cell line H134AD, maintained in 1700 nM DOX, and the parental cell line H134 were originally established by Dr HJ Broxterman (Department of Medical Oncology, Free University Hospital, Amsterdam, the Netherlands) and kindly provided by Dr H Heyligen (Department of Monoclonal Antibodies, Dr L Willems Institute, Diepenbeek, Belgium). The adherent cultures of all cell lines were maintained in RPMI-1640 medium (Gibco, Paisley, UK) supplemented with 10% bovine calf serum (HyClone Laboratories, Logan, UT, USA) at 37°C in 5% carbon dioxide/95% air. Other characteristics of the cell lines have been reported elsewhere (Scheper *et al*., 1988, 1993; Maillet *et al*., 1993). All studies were carried out in early confluent cultures on plastic tissue culture plates (Greiner, Frickenhausen, Germany).

*Immunostaining*

To perform immunostaining, cells were fixed with 4% paraformaldehyde and permeabilised with 0.1% saponin. P-gp, LRP and MRP were stained respectively with monoclonal antibodies JSB-1 (Scheper *et al*., 1988), LRP-56 (Scheper *et al*., 1993) and MRPm6 (Flens *et al*., 1994) at dilution 1:100 overnight at 4°C. All antibodies were provided by Dr RJ Scheper. The secondary FITC-conjugated antibodies were isotype specific and purchased from Seralab (Sussex, UK) and Southern Biotechnology Associates (Birmingham, AL, USA). The secondary antibodies were used at a 1:200 dilution for 3 h at 20°C. The level of immunostaining was estimated by flow cytometry as the mean fluorescence channel in a FACS 4 (Becton Dickinson, Mountain View, CA, USA).

*Cytotoxicity assays*

The drug cytotoxicity ($IC_{50}$) at continuous exposure was estimated by a 5 day MTT assay as described previously (Delvaeye *et al*., 1993). The surviving fraction (SF) of cells exposed to DOX for 4 h was assessed by a modified MTT serial dilution assay carried out as follows. Confluent cultures of PSN1 and PSN1/ADR cells were exposed to DOX at concentrations indicated in the legends, washed with PBS and harvested by trypsinisation. Six serial dilutions (0.5 log-fold) of the cell suspension, starting from $10^4$ cells per well, were reseeded into a 96-well plate and incubated for 5 days. The MTT assay was performed for 3 h at 37°C in 80 $\mu l$ of fresh medium containing 0.5 mg $ml^{-1}$ MTT [3-(4,5-dimethylthiazol-2-yl)-2,5-diphenyltetrazolium bromide]. The reaction was stopped by careful addition of 200 $\mu l$ of DMSO − 0.05 M HCl. The formazan crystals were dissolved in the lower layer of DMSO during 5−10 min at 37°C. Afterwards the DMSO and medium layers were mixed by repeated pipetting and the absorbency was measured at 540 nm. The surviving fraction (SF) was calculated as the dilution factor of the control that produced the same optical density as the treated samples.

*Doxorubicin accumulation*

Cell cultures were exposed to 1 $\mu g\ ml^{-1}$ DOX alone or with modulators at specified concentrations for 4 h at 37°C and washed with PBS on ice. The incubation period of 4 h was selected in preliminary experiments as the end point, corresponding to a steady-state cellular drug concentration. The cellular DOX content was estimated by a fluorometric assay in a 50% ethanol/0.05 N HCl extract and normalised to DNA (ng DOX $\mu g^{-1}$ DNA) as described previously (Delvaeye *et al*., 1993). We preferred to use the DNA content rather than the cell count to normalise the DOX uptake because the adherent cells were fixed by the ethanol extraction procedure and therefore difficult to resuspend.

*Doxorubicin retention*

The cell cultures were exposed to 10 $\mu g\ ml^{-1}$ DOX for 1 h at 37°C, washed with PBS and reincubated for 2 h at 37°C in drug-free medium with modulators at the indicated concentrations. The incubation time of 2 h was selected in preliminary experiments as the end point providing a reasonable divergence of the drug retention−time curves for PSN1 and PSN1/ADR cells. Afterwards, the cells were washed with PBS on ice and processed for analysis of the cellular DOX content as in the DOX accumulation experiments. The cellular DOX content after a 1 h uptake was taken as 100%.

*Rhodamine 123 uptake*

Cell cultures were exposed to 0.2 $\mu g\ ml^{-1}$ rhodamine 123 for 1 h at 37°C, washed with cold PBS and trypsinised. The intracellular rhodamine 123 content was analysed by flow cytometry in a FACS 4.

*ECL Western immunoblotting*

Cells were lysed in a buffer containing 137 mM sodium chloride, 20 mM Tris-HCl (pH 8.0), 1% triton X-100, 0.5% deoxycholate, 0.1% SDS, 1 mM phenylmethylsulphonyl fluoride, 1 $\mu g\ ml^{-1}$ aprotinin and 1 $\mu g\ ml^{-1}$ pepstatin. The lysates were sonicated, clarified for 20 min at 13 000 *g* and mixed 1:4 (vol/vol) with a standard SDS/2-mercaptoethanol-containing sample buffer. The protein extracts from $10^5$ cells were resolved in an SDS/PAGE using a 7.5% resolving gel (1.5 mm thickness), and electrophoretically transferred onto Hybond-C super nitrocellulose membrane (Amersham, Buckinghamshire, UK). The membranes were stained for 1 h at 20°C with the primary monoclonal antibodies to P-gp (JSB-1, 1:300) and MRP (MRPm6, 1:100). The blots were analysed by an immunoperoxidase-based ECL technique (Amersham) according to the manufacturer's protocol.

### *Statistics*

All assays were repeated 3–6 times. Data are expressed as arithmetical means (points) with corresponding standard deviations (bars).

### Results

### *Immunocytochemical and pharmacological phenotype of PSN1/ADR cells*

PSN1/ADR cells selected in 17–51 nM DOX showed the activation of LRP but not MRP as assessed by FACS and shown for the PSN1/ADR7.3 subline in Figure 1. Further selection in 170 nM DOX (sublines PSN1/ADR17 and PSN1/ADR33) led to the activation of MRP, whereas the LRP level remained stable. The acquisition of drug resistance to 170 nM DOX was caused by DOX accumulation defects reversible by 10 μM verapamil, 320 μM BFA and 10 μM fluoroaluminate. In the parental PSN1 cells the DOX uptake was increased only by verapamil, whereas BFA at 10 μM caused a small but distinct protective effect resulting in a 20±9.0% decrease ($P<0.05$) in cellular DOX content. The immunoblot data on MRP and P-gp expression were in agreement with FACS analysis, and the presence of a low but detectable level of MRP in the parental PSN1 cells was observed (Figure 2). MRP immunostaining in PSN1 cells was clearly positive upon ECL overexposure (3 min instead of 15 s), while P-gp remained undetectable (data not shown). PSN1/ADR96 cells, selected in 510 nM DOX, retained a P-gp-negative phenotype while MRP expression was reduced. The DOX accumulation defects remained sensitive to verapamil, BFA and fluoroaluminate with a maximal response to chemomodifiers at a 4–8 h exposure, corresponding to the steady-state level of cellular DOX concentrations (Figure 3). These kinetics were similar in all of the PSN1/ADR sublines (data not shown).

The H134AD cells originally selected in 1700 nM DOX overexpressed P-gp (Figure 2) in agreement with literature (Scheper *et al.*, 1988). The latter cells were used in this study as a model of P-gp-mediated DOX resistance.

### *Flow cytometry of the rhodamine 123 uptake*

The fluorescent dye rhodamine 123 is thought to be a relevant probe for the P-gp efflux pump and provides a sensitive functional assay, alternative to immunostaining approaches (Gottesman and Pastan, 1993). Indeed, the

**Figure 1** Evolution of immunocytochemical phenotype (**a**) and doxorubicin accumulation defects (**b**) in the human pancreatic tumour PSN1/ADR cells during the course of stepwise selection in 17–170 nM doxorubicin. (**a**) P-gp, MRP and LRP expression was measured by FACS, using the monoclonal antibodies JSB-1, MRPm6 and LRP-56 respectively. (**b**) The doxorubicin accumulation defects were probed with brefeldin A, fluoroaluminate and verapamil in a 4 h drug accumulation assay. The cells were exposed to 1.0 μg ml$^{-1}$ doxorubicin alone or in combination with modulators at the indicated concentrations and the cellular doxorubicin content was normalised to DNA. Doxorubicin resistance index 1 corresponds to the parental PSN1 cells. The resistant sublines are indicated by their resistance indices (7.3, 17 and 33) and are named later PSN1/ADR7.3, PSN1/ADR17 and PSN1/ADR33.

**Figure 2** Western blot analysis of the MRP (**a**) and P-gp (**b**) expression in H134AD and PSN1 cells and the PSN1/ADR sublines named according to their doxorubicin resistance index. The protein samples extracted from $10^5$ cells were resolved in a 7.5% polyacrylamide gel containing 0.1% SDS. The blots were probed for P-gp and MRP with the monoclonal antibodies JSB-1 and MRPm6 respectively and analysed by an immunoperoxidase-based ECL technique.

**Figure 3** The kinetics of doxorubicin accumulation in PSN1/ADR96 cells. The cellular doxorubicin content was measured during exposure to 1 μg ml$^{-1}$ doxorubicin alone (- - -) or in the presence of 10 μM fluoroaluminate (□), 10 μM verapamil (○) or 320 μM brefeldin A (△).

**Figure 4** Flow cytometry of the rhodamine 123 uptake in H134AD cells, in the PSN1/ADR sublines and in the parental cells. Representation of a typical cell population analysis after exposure of cell cultures to 0.2 $\mu$g ml$^{-1}$ of rhodamine 123 for 1 h. The position of all blanks (not exposed to rhodamine 123) was adjusted to that of the H134 cells.

rhodamine 123 uptake was significantly reduced in the P-gp-positive H134AD cells relative to the H134 cells (Figure 4). To compare the FACS data, all blank samples (not exposed to rhodamine 123) were adjusted to that of H134 cells by tuning the amplification of the fluorescence signal. In contrast to H134AD cells the rhodamine 123 signal in the MRP-positive PSN1/ADR sublines was close to that of the parental cell line PSN1, although for the PSN1/ADR33 cells we observed a somewhat decreased rhodamine 123 uptake. This comparison confirms the low activity of the P-gp pump in PSN1/ADR cells as expected from the immunostaining experiments (Figures 1 and 2).

### *Drug cross-resistance in PSN1/ADR cells*

The PSN1/ADR33 cells selected in 170 nM DOX demonstrated an MDR phenotype with classical cross-resistance to natural cytotoxins including etoposide, vincristine and actinomycin D (Table I). The resistance index (7.6- to 39-fold) between the PSN1/ADR33 and PSN1 cells could not be attributed to any differences in growth rate since both cell lines possessed the same doubling time of 21 ± 2 h. A much lower resistance index was observed for colchicine and rhodamine 123, being respectively 4.3 and 3.9. There was also a weak cross-resistance (3.1-fold) to cisplatin, a drug that is not normally involved in MDR. BFA and genistein did not show cross-resistance to DOX and revealed spectacular difference in cytotoxicity at continuous exposure in contrast to growth inhibition effects at a short-term exposure (see below).

### *Effects of brefeldin A and verapamil on doxorubicin cytotoxicity*

The high cytotoxicity of BFA at continuous exposure did not allow us to conduct chemosensitising experiments using a classical MTT assay. Therefore, confluent cultures of PSN1 and PSN1/ADR33 cells were exposed to DOX and chemomodifiers for 4 h and afterwards the cells were reseeded to assess the cell survival (Table II). As in the pharmacological studies, BFA demonstrated both a sensitising or a protective effect, depending on the drug resistance level. In the PSN1 cells BFA at 10–32 $\mu$M reduced the DOX cytotoxicity by 2.1 to 2.4-fold. In the PSN1/ADR33 cells, BFA at a maximal non-cytotoxic concentration of 320 $\mu$M ($SF > 0.8$ for BFA alone) increased the DOX cytotoxicity by 2.5-fold. In contrast to BFA verapamil sensitised both the PSN1 and PSN1/ADR33 cells with a preferential reversal effect in the latter cells. The sensitisation index of verapamil and BFA in the PSN1/ADR33 cells was approximately equal and rather modest compared with the level of acquired resistance. Genistein at active modulatory concentrations of 100–320 $\mu$M demonstrated a delayed cytotoxicity ($SF < 0.8$ alone) and therefore was not assessed as a reverser of DOX resistance.

**Table I** Drug cross-resistance in PSN1 and PSN1/ADR33 cells

| *Drugs* | *IC*$_{50}$ ($\mu$M)[a] *PSN1* | *PSN1/ADR33* | *RI*[b] |
|---|---|---|---|
| Etoposide | 0.17 ± 0.026 | 6.7 ± 1.5 | 39 |
| Doxorubicin | 0.0082 ± 0.00055 | 0.27 ± 0.036 | 33 |
| Daunorubicin | 0.0094 ± 0.00063 | 0.11 ± 0.045 | 12 |
| Vincristine | 0.00079 ± 0.000056 | 0.0076 ± 0.0036 | 9.6 |
| Actinomycin D | 0.00025 ± 0.000096 | 0.0019 ± 0.00041 | 7.6 |
| Colchicine | 0.0097 ± 0.0021 | 0.042 ± 0.0076 | 4.3 |
| Rhodamine 123 | 0.70 ± 0.11 | 2.7 ± 0.29 | 3.9 |
| Cisplatin | 0.14 ± 0.015 | 0.43 ± 0.15 | 3.1 |
| Taxol | 0.0039 ± 0.00059 | 0.0067 ± 0.0039 | 1.7 |
| Mitomycin C | 0.033 ± 0.0035 | 0.053 ± 0.003 | 1.6 |
| Fluoroaluminate | 0.77 ± 0.22 | 1.1 ± 0.047 | 1.4 |
| 5-Fluorouracil | 0.73 ± 0.11 | 0.80 ± 0.39 | 1.1 |
| Nocodazole | 0.049 ± 0.0012 | 0.054 ± 0.0021 | 1.1 |
| Amsacrine | 4.6 ± 0.75 | 4.8 ± 0.23 | 1.0 |
| Genistein | 36 ± 4.2 | 37 ± 3.5 | 1.0 |
| Verapamil | 71 ± 1.7 | 70 ± 6.6 | 0.99 |
| Brefeldin A | 0.064 ± 0.011 | 0.060 ± 0.012 | 0.94 |

[a]$IC_{50}$, drug cytotoxicity was determined at continuous exposure using a 5 day MTT assay. [b]RI, the resistance index, was calculated by dividing the $IC_{50}$ value of PSN1/ADR33 cells (passage 90) by the $IC_{50}$ value of the parental PSN1 cells.

### *Doxorubicin retention in the PSN1/ADR and H134AD cells*

The mechanisms of DOX extrusion in the MRP-positive PSN1/ADR33 cells and in the P-gp-positive H134AD cells were probed by diverse inhibitors of drug transporters and vesicular traffic. To compare a modulatory potency, agents were tested in the range of at least 10-fold dilutions using a fixed 2 h time point in the DOX retention assay (Figure 5). Genistein and BFA decreased the DOX efflux in the PSN1/ADR33 but not H134AD cells with identical specificity and molar inhibitory potency. Fluoroaluminate and nocodazole were also potent inhibitors of the DOX efflux only in the

**Table II** Effect of brefeldin A and verapamil on doxorubicin cytotoxicity in PSN1 and PSN1/ADR33 cells.

| *Treatment* | *PSN1/ADR33* *SF*[a] | *SI*[b] | *PSN1* *SF* | *SI* |
|---|---|---|---|---|
| DOX alone | 0.43 ± 0.065[c] | 1.0 | 0.084 ± 0.018 | 1.0 |
| DOX + 10 $\mu$M verapamil | 0.19 ± 0.032[c] | 2.3 | 0.056 ± 0.014[c] | 1.5 |
| DOX + 10 $\mu$M BFA | 0.36 ± 0.070 | 1.2 | 0.20 ± 0.066[c] | 0.42 |
| DOX + 32 $\mu$M BFA | 0.28 ± 0.051[c] | 1.5 | 0.18 ± 0.044[c] | 0.47 |
| DOX + 100 $\mu$M BFA | 0.23 ± 0.027[c] | 1.9 | 0.15 ± 0.027[c] | 0.56 |
| DOX + 320 $\mu$M BFA | 0.17 ± 0.031[c] | 2.5 | 0.12 ± 0.018[c] | 0.70 |
| 10 $\mu$M verapamil alone | 0.98 ± 0.051 | | 0.99 ± 0.072 | |
| 320 $\mu$M BFA alone | 0.88 ± 0.12 | | 0.93 ± 0.093 | |

[a]SF, survival fraction of cells after 4 h drug treatment was estimated by the MTT serial dilution assay. PSN1 and PSN1/ADR33 cells (passage 90) were exposed to doxorubicin alone at 0.1 and 1.0 $\mu$g ml$^{-1}$ respectively or in combination with brefeldin A or verapamil at the specified concentrations. [b]SI, the sensitisation index, was calculated by dividing the SF for doxorubicin alone by the SF for doxorubicin plus chemomodifier. [c]$P < 0.01$ compared with doxorubicin alone.

PSN1/ADR33 cells. The effects of fluoroaluminate and genistein should be interpreted with caution since they are somewhat cytotoxic at modulatory concentrations (data not shown). It is worth noting that verapamil was a rather non-specific inhibitor of the DOX efflux in both drug-resistant cell lines, and was unexpectedly more active in the MRP-positive PSN1/ADR33 cells. However, the restoration of DOX retention by verapamil in the latter cells did not result in a substantial chemosensitisation, as shown in Table II. The DOX retention levels in the parental PSN1 and H134 cells were 63±6.2 and 58±4.1% respectively. The modulatory

**Figure 5** Modulation of doxorubicin efflux in PSN1/ADR33 and H134AD cells by brefeldin A (○, **a**) genistein (●, **a**), fluoroaluminate (**b**), nocodazole (**c**) and verapamil (**d**). Cell cultures were exposed to $10\,\mu g\,ml^{-1}$ doxorubicin for 1 h, washed with PBS and reincubated for 2 h in drug-free medium with or without a modulator. The cellular doxorubicin content is expressed as a percentage relative to the amount after 1 h uptake. (- - -), Level of doxorubicin retention in the absence of chemomodifiers.

effects of all inhibitors in these cells were below 25% and are not presented here. The comparison of DOX accumulation – retention patterns in the PSN1 and PSN1/ADR33 cells clearly demonstrated an accelerated drug extrusion as the mechanism of reduced drug accumulation and acquired DOX resistance.

### Discussion

Selection of the human pancreatic tumour cell line PSN1/ADR in 17–510 nM DOX activated various MDR markers and mechanisms of drug resistance without inducing a detectable level of P-gp. In both flow cytometry and ECL Western blot immunostaining the PSN1/ADR33 cells adapted to 170 nM DOX at intermittent exposure displayed a rise in MRP only. The P-gp efflux pump remained undetectable by the rhodamine 123 functional assay (Gottesman and Pastan, 1993). The DOX accumulation–retention defects in the MRP-positive PSN1/ADR33 cells were reversible by the vesicle traffic inhibitors BFA and fluoroaluminate. In contrast, in the P-gp-overexpressing H134AD cells adapted to 1700 nM DOX at continuous exposure (Scheper *et al.*, 1988), rhodamine 123 uptake was significantly decreased, while both BFA and fluoroaluminate failed to inhibit DOX extrusion. Therefore, our data suggest that the BFA/fluoroaluminate-sensitive mechanism of DOX extrusion plays a role in low-level DOX resistance associated with the MRP transporter, but may be attenuated in highly resistant clones possessing the more efficient P-gp efflux pump. A prevalent role of MRP over P-gp in early steps of MDR acquisition emerged in recent literature (Eijdems *et al.*, 1995). Another early MDR marker found in P-gp-negative MDR clones seems to be LRP (Scheper *et al.*, 1993), which we observed already in PSN1/ADR7.3 cells selected in 17–51 nM DOX. While the function of LRP is not known, the MRP transporter can confer broad-spectrum MDR (Cole *et al.*, 1994), and is likely to underlie the MDR phenotype of PSN1/ADR33 cells. The MRP-positive PSN1/ADR33 cells exhibit an MDR spectrum similar to P-gp-mediated MDR (Gottesman and Pastan, 1993) except for an unusual cross-resistance to cisplatin, reported also for the P-gp-negative N592/DX cells (Supino *et al.*, 1993).

The reversal effects of chemomodifiers in 190 kDa/MRP-overexpressing cells appear to be reduced and more variable than in P-gp-positive MDR cells (McGrath *et al.*, 1989; Barrand *et al.*, 1993; Versantvoort *et al.*, 1993; Cole *et al.*, 1994). In our experiments with MRP-positive PSN1/ADR33 cells the reversal effect of verapamil on DOX resistance was modest relative to the magnitude of acquired resistance. In the parental PSN1 cells verapamil also demonstrated sensitising effects, whereas BFA caused chemoprotection. The latter effect has been previously described in L1210 cells (Vichi and Tritton, 1993). Important findings are that BFA possesses a reversing activity similar to that of verapamil in PSN1/ADR33 cells, and that concentrations of BFA up to 320 $\mu$M are not directly cytotoxic after a time period of 2–4 h. In short-term experiments with the P-gp-negative MDR COR-L23/R cells BFA was reported to be cytotoxic already at 40 $\mu$M and was therefore not regarded as a potential MDR reverser, although it did increase the cellular drug accumulation and the nuclear–cytoplasmic ratio for anthracyclines (Rhodes *et al.*, 1994). The reason for this discrepancy remains to be elucidated, but we cannot exclude that BFA toxicity is cell line dependent. It is also possible that BFA provoked more cell detachment in the sparse cultures used by Rhodes *et al.* (1994) than in the confluent cultures in our protocol.

To provide new insights into the multifactorial nature of MDR more functional probes addressed to non-P-gp-mediated drug transport are needed. Genistein was recently proposed as a probe for non-P-gp-related drug accumulation defects, although cytotoxicity at the modulatory concentrations of 100–300 $\mu$M was observed (Versantvoort *et al*, 1993). Genistein appears to be a competitive inhibitor of anthracycline transport attributed to the MRP efflux pump

(Versantvoort et al., 1994). This mechanism could account for the genistein-sensitive drug accumulation defects in the MRP-positive PSN1/ADR33 cells presented here, and in a variety of MRP-positive MDR cell lines (GLC4/ADR, SW-1573/2R120, HT1080/DR4, HL60/ADR) described elsewhere (Versantvoort et al., 1993). However, the reversal of DOX resistance by genistein was reported also for the MRP-negative K562/TPA cells, thereby indicating the existence of target(s) different from MRP (Takeda et al., 1994). Additionally, ATP depletion (Versantvoort et al., 1994) and the inhibition of the G protein-linked secretory pathway (Duan et al., 1994) were observed in cells exposed to genistein at high concentrations.

BFA has not yet been studied extensively as a chemomodifier of DOX resistance. Our data demonstrates that BFA is capable of complete discrimination between ADR extrusion pathways in MRP-positive PSN1/ADR33 and P-gp-positive H134AD cells. Its specificity and potency seem to be close to those of genistein. The reversal of DOX resistance by BFA in PSN1/ADR33 cells is optimal at 100–320 $\mu$M, whereas in cells not selected for MDR the inhibition of vesicular traffic by BFA occurs already at 10 $\mu$M (Klausner et al., 1992). This discrepancy raises the question of whether BFA is a specific enough probe to implicate drug-trapping vesicles in the mechanism of DOX extrusion. A striking similarity between the inhibitory potency of BFA and genistein in the MRP-positive PSN1/ADR33 cells would suggest that BFA, by analogy to genistein (and verapamil), might be a competitive inhibitor of the MRP efflux pump. This assumption is in line with the concomitant activation of MRP and the outward DOX transport sensitive to genistein, BFA and verapamil. However, the inhibitory effects of verapamil can also be attributed to membrane traffic perturbations (Sehested et al., 1987). In addition, the above explanation is not consistent with the opposite effects of BFA and verapamil on DOX cytotoxicity in parental PSN1 cells that displayed a low but detectable level of MRP. Secondly, DOX extrusion in PSN1/ADR33 cells was sensitive to fluoroaluminate, an agent which disrupts GTP-dependent vesicular transport (Barr et al., 1991), and is unlikely to exert preferential inhibition of MRP compared with P-gp when its interference with ATP-binding sites is supposed. Thirdly, nocodazole, an agent that can block the vesicular transport by microtubule depolymerisation (Breitfeld et al., 1990), inhibited substantially the DOX extrusion in the PSN1/ADR33 but not H134AD cells. Finally, PSN1/ADR96 cells adapted to 510 nM DOX retained BFA-sensitive DOX accumulation defects whereas MRP declined. Hence, it seems unlikely that BFA would target solely the MRP transporter in this cell subline.

In the extensive literature now available on BFA its target is thought to be the ARF GDP/GTP exchange factor, specifically ascribed to G protein-controlled vesicular traffic (Donaldson et al., 1992; Klausner et al., 1992). Interestingly, the activated pool of G proteins in a GTP-bound form was shown to resist low concentrations of BFA (Klausner et al., 1992; Ktistakis et al., 1992). Therefore, elevated modulatory concentrations of BFA in PSN1/ADR33 cells might indicate an enriched pool of activated G proteins that trigger vesicular exocytosis. In the view of this hypothesis, BFA-sensitive vesicular transport could particularly sustain DOX resistance, since lipophilic and weakly basic anthracyclines can be trapped inside vesicular structures (Gervasoni et al., 1991; Coley et al., 1993). Future studies should clarify whether the model of vesicle-mediated drug transport is relevant to the BFA/fluoroaluminate-sensitive mechanism of DOX extrusion or whether BFA is an inhibitor of MRP. In this paper, we focused primarily on reversal properties of G protein-targeting agents in an effort to provide a rationale for searching for novel functional probes of MDR not associated with the P-gp transporter. Our preliminary tests have shown a clear correlation ($r > 0.9$) between the BFA reversal effects and DOX resistance in a panel of P-gp-negative human pancreatic tumour cell lines (Verovski et al., 1994). This is further support for BFA as an appropriate tool to dissect the mechanisms of DOX resistance not associated with the P-gp efflux pump.

### Abbreviations

MDR, multidrug resistance; P-gp, P-glycoprotein; MRP, multidrug resistance-associated protein; LRP, lung resistance-related protein; ABC, ATP-binding cassette; G proteins, GTP-binding proteins; DOX, doxorubicin; BFA, brefeldin A; ARF, ADP-ribosylation factor; SF, survival fraction.

**Acknowledgements**
This research was funded by grants nos. 3.0036.94 and G.0064.95 from the Nationaal Fonds voor Wetenschappelijk Onderzoek (NFWO) and Sportvereniging tegen Kanker. The authors wish to thank Mrs C Monsaert and Mr L De Lange for valuable technical support. We are grateful to Professor Dr M Mareel for critical review. We also acknowledge Professor Dr G Klöppel, who kindly provided the human pancreatic tumour cell line PSN1, and Dr H Broxterman and Dr H Heyligen from whom we received the H134 and H134AD cell lines.

### References

BARR FA, LEYTE A, MOLLNER S, PFEUFFER T, TOOZE SA AND HUTTNER WB. (1991). Trimeric G-proteins of the trans-Golgi network are involved in the formation of constitutive secretory vesicles and immature secretory granules. *FEBS Lett.*, **294**, 239–243.

BARRAND MA, RHODES T, CENTER MS AND TWENTYMAN PR. (1993). Chemosensitisation and drug accumulation effects of cyclosporin A, PSC-833 and verapamil in human MDR large cell lung cancer cells expressing a 190 K membrane protein distinct from P-glycoprotein. *Br. J. Cancer*, **29**, 408–415.

BECK WT. (1987). The cell biology of multiple drug resistance. *Biochem. Pharmacol.*, **36**, 2879–2887.

BREITFELD PP, MCKINNON WC AND MOSTOV KE. (1990). Effect of nocodazole on vesicular traffic to the apical and basolateral surfaces of polarized MDCK cells. *J. Cell Biol.*, **111**, 2365–2373.

COLE SPC, BHARDWAJ G, GERLACH JH, MACKIE JE, GRANT CE, ALMQUIST KC, STEWART AJ, KURZ EU, DUNCAN AMV AND DEELEY RG. (1992). Overexpression of a transporter gene in a multidrug-resistant human lung cancer cell line. *Science*, **258**, 1650–1654.

COLE SPC, SPARKS KE, FRASER K, LOE DW, GRANT CE, WILSON GM AND DEELEY RG. (1994). Pharmacological characterization of multidrug resistant MRP-transfected human tumor cells. *Cancer Res.*, **54**, 5902–5910.

COLEY HM, AMOS WB, TWENTYMAN PR AND WORKMAN P. (1993). Examination by laser scanning confocal fluorescence imaging microscopy of the subcellular localisation of anthracyclines in parent and multidrug resistant cell lines. *Br. J. Cancer*, **67**, 1316–1323.

DELVAEYE M, VEROVSKI V, DE NEVE W AND STORME G. (1993). DNA breakage, cytotoxicity, drug accumulation and retention in two human ovarian tumor cell lines AZ224 and AZ364 treated with adriamycin, modulated by verapamil. *Anticancer Res.*, **13**, 1533–1538.

DONALDSON JG, FINAZZI D AND KLAUSNER RD. (1992). Brefeldin A inhibits Golgi membrane-catalysed exchange of guanine nucleotide onto ARF protein. *Nature*, **360**, 350–352.

DUAN RD, WAGNER ACC, YULE DI AND WILLIAMS JA. (1994). Multiple inhibitory effects of genistein on stimulus-secretion coupling in rat pancreatic acini. *Am. J. Physiol.*, **266**, G303–G310.

EIJDEMS EWHM, ZAMAN GJR, DE HAAS M, VERSANTVOORT CHM, FLENS MJ, SCHEPER RJ, KAMST E, BORST P AND BAAS F. (1995). Altered MRP is associated with multidrug resistance and reduced drug accumulation in human SW-1573 cells. *Br. J. Cancer,* **72,** 298–306.

FLENS MJ, IZQUIERDO MA, SCHEFFER GL, FRITZ JM, MEIJER CJLM, SCHEPER RJ AND ZAMAN GJR. (1994). Immunochemical detection of the multidrug resistance-associated protein MRP in human multidrug-resistant tumor cells by monoclonal antibodies. *Cancer Res.*, **54,** 4557–4563.

GERVASONI Jr JE, FIELDS SZ, KRISHNA S, BAKER MA, ROSADO M, THURAISAMY K, HINDENBURG AA AND TAUB RN. (1991). Subcellular distribution of daunorubicin in P-glycoprotein-positive and -negative drug-resistant cell lines using laser-assisted confocal microscopy. *Cancer Res.*, **51,** 4955–4963.

GOTTESMAN MM AND PASTAN I. (1993). Biochemistry of multidrug resistance mediated by the multidrug transporter. *Annu. Rev. Biochem.*, **62,** 385–427.

KLAUSNER RD, DONALDSON JG AND LIPPINCOTT-SCHWARTZ J. (1992). Brefeldin A: insights into the control of membrane traffic and organelle structure. *J. Cell Biol.*, **116,** 1071–1080.

KTISTAKIS NT, LINDER ME AND ROTH MG. (1992). Action of brefeldin A blocked by activation of a pertussis-toxin-sensitive G protein. *Nature*, **356,** 344–346.

MCGRATH T, LATOUD C, ARNOLD ST, SAFA AR, FELSTED RL AND CENTER MS. (1989). Mechanisms of multidrug resistance in HL60 cells. Analysis of resistance associated membrane proteins and levels of MDR gene expression. *Biochem. Pharmacol.*, **38,** 3611–3619.

MAILLET B, DE GREVE J, LEMOINE N, KALTHOFF H, SCHMIEGEL W AND KLÖPPEL G. (1993). Phenotypical differentiation and genetic alterations in human pancreatic carcinoma cell lines. *Int. J. Pancreatol.*, **14,** 72–75.

MARQUARDT D AND CENTER MS. (1992). Drug transport mechanisms in HL60 cells isolated for resistance to adriamycin: evidence for nuclear drug accumulation and redistribution in resistant cells. *Cancer Res.*, **52,** 3157–3163.

RHODES T, BARRAND MA AND TWENTYMAN PR. (1994). Modification by brefeldin A, bafilomycin $A_1$, and 7-chloro-4-nitrobenz-2-oxa-1,3-diazole (NBD) of cellular accumulation and intracellular distribution of anthracyclines in the non-P-glycoprotein-mediated multidrug-resistant cell line COR-L23/R. *Br. J. Cancer*, **70,** 60–66.

SCHEPER RJ, BULTE JWM, BRAKKEE JGP, QUAK JJ, VAN DER SCHOOT E, BALM AJM, MEIJER CJLM, BROXTERMAN HJ, KUIPER CM, LANKELMA J AND PINEDO HM. (1988). Monoclonal antibody JSB-1 detects a highly conserved epitope on the P-glycoprotein associated with multi-drug-resistance. *Int. J. Cancer*, **42,** 389–394.

SCHEPER RJ, BROXTERMAN HJ, SCHEFFER GL, KAAIJK P, DALTON WD, VAN HEIJNINGEN THM, VAN KALKEN CK, SLOVAK ML, DE VRIES EGE, VAN DER VALK P, MEIJER CJLM AND PINEDO HM. (1993). Overexpression of a $M_r$ *110,000* vesicular protein in non-P-glycoprotein-mediated multidrug resistance. *Cancer Res.*, **53,** 1475–1479.

SEHESTED M, SKOVSGAARD T, VAN DEURS B AND WINTHER-NIELSEN H. (1987). Increased plasma membrane traffic in daunorubicin resistant P388 leukaemic cells. Effect of daunorubicin and verapamil. *Br. J. Cancer*, **56,** 747–751.

SUPINO R, BINASCHI M, CAPRANICO G, GAMBETTA RA, PROSPERI E, SALA E AND ZUNINO F. (1993). A study of cross-resistance pattern and expression of molecular markers of multidrug resistance in a human small-cell lung-cancer cell line selected with doxorubicin. *Int. J. Cancer*, **54,** 309–314.

TAKEDA Y, NISHIO K, NIITANI H AND SAIJO N. (1994). Reversal of multidrug resistance by tyrosine-kinase inhibitors in a non-P-glycoprotein-mediated multidrug-resistant cell line. *Int. J. Cancer*, **57,** 229–239.

TWENTYMAN PR, RHODES T AND RAYNER S. (1994). A comparison of rhodamine 123 accumulation and efflux in cells with P-glycoprotein-mediated and MRP-associated multidrug resistance phenotypes. *Eur. J. Cancer*, **9,** 1360–1369.

VEROVSKI V, DELVAEYE M, VAN DEN BERGE D, DE NEVE W AND STORME G. (1994). Reversing potential of brefeldin A in non-P-glycoprotein mediated cellular resistance to adriamycin. 1st International Conference on reversal of multidrug resistance in cancer (abstr.). *Anti-Cancer Drugs*, **5** (suppl. **1**), 31–32.

VERSANTVOORT CHM, SCHUURHUIS GJ, PINEDO HM, EEKMAN CA, KUIPER CM, LANKELMA J AND BROXTERMAN HJ. (1993). Genistein modulates the decreased drug accumulation in non-P-glycoprotein-mediated multidrug-resistant tumour cells. *Br. J. Cancer*, **68,** 939–946.

VERSANTVOORT CHM, BROXTERMAN HJ, LANKELMA J, FELLER N AND PINEDO HM. (1994). Competitive inhibition by genistein and ATP dependence of daunorubicin transport in intact MRP overexpressing human small cell lung cancer cells. *Biochem. Pharmacol.*, **6,** 1129–1136.

VICHI PJ AND TRITTON TR. (1993). Protection from adriamycin cytotoxicity in L1210 cells by brefeldin A. *Cancer Res.*, **53,** 5237–5243.